# Associations of perfectionism dimensions with perceived stress and anxiety symptoms among Chinese undergraduates in sport-related majors: a multi-site cross-sectional study

**DOI:** 10.3389/fpsyg.2026.1910894

**Published:** 2026-08-17

**Authors:** Xiangqian Xu, Georgiy Korobeynikov, Oleksandr Pryimakov, Lesia Korobeinnikova, Fatong Wan, Wei Han, Zan Li

**Affiliations:** 1 Institute of Competitive Sport, Shandong Sport University, Rizhao, China; 2 Uzbek State University of Physical Education and Sport, Chirchik, Uzbekistan; 3 National University of Ukraine on Physical Education and Sport, Kyiv, Ukraine; 4 Faculty of Physical Culture and Health Promotion, Szczecin University, Szczecin, Poland; 5 Institute of Competitive Sport, Tianjin Sport University, Tianjin, China

**Keywords:** anxiety symptoms, path analysis, perceived stress, perfectionism dimensions, sport-related majors, university students

## Abstract

**Background:**

Undergraduates in sport-related majors may face overlapping academic, practical, physical-performance, and, for some students, training and competition demands. Perfectionism dimensions may shape how these demands are appraised.

**Objective:**

To examine the associations of High Standards, Order, and Discrepancy with perceived stress and anxiety symptoms among Chinese undergraduates in sport-related majors, including cross-sectional indirect associations through empirically supported perceived-stress components.

**Methods:**

A multi-site cross-sectional survey included 705 full-time undergraduates from 19 institutions across mainland China. Participants completed the Chinese Almost Perfect Scale-Revised, a perceived-stress measure contextually adapted from the Chinese PSS-14, and the GAD-7. Confirmatory factor analyses guided score representation. Because the APS-R latent models fitted poorly and High Standards and Order overlapped substantially, the three subscale scores were modeled as observed predictors. The adapted PSS-14 was represented by low perceived coping and negative stress appraisal rather than a total score. Direct and indirect associations were examined using robust and bootstrap procedures, with covariate-adjusted and hybrid SEM sensitivity analyses.

**Results:**

Discrepancy showed the clearest adverse pattern across perceived stress and anxiety symptoms. Order was associated with lower scores on both perceived-stress components, whereas High Standards showed only a limited association with low perceived coping. Neither High Standards nor Order showed a direct association with anxiety symptoms. Negative stress appraisal was more closely related to anxiety than low perceived coping. The indirect-association pattern was evident for Discrepancy and Order but not for High Standards, and the overall interpretation was unchanged in the sensitivity analyses.

**Conclusion:**

Persistent perceptions of falling short of personally important standards may be more relevant to anxiety symptoms than demanding standards alone. The findings also suggest that negative appraisal of demands may be more closely aligned with anxiety than perceived coping difficulties. Interpretation should remain cautious because the APS-R dimensions were not fully distinct, the adapted PSS-14 components were aligned with item wording, and the cross-sectional design does not establish temporal or causal relationships.

## Introduction

University student mental health has become an important educational and public health concern in China. In 2024, higher education enrollment reached 48.46 million, including approximately 20.86 million regular undergraduate students (Ministry of Education of the People’s Republic of China, 2025a, 2025b). Students enrolled in sport-related majors constitute a distinct educational group within this population (Ministry of Education of the People’s Republic of China, 2025a, 2025b). Depending on the program, their education may combine academic coursework with practical skill assessment, physical-performance requirements, and organized training or competition. Academic and performance-based expectations may therefore converge within the same educational setting.

Anxiety symptoms are important in university populations because they are associated with emotional maladjustment, role impairment, and reduced quality of life (Ahmed et al., 2023; Alonso et al., 2018; Beiter et al., 2015). Evidence from related sport and educational settings helps clarify the demands that may be relevant to students in sport-related programs. Kosmidou et al. (2025) reported that practical examinations among physical education and sport students may be accompanied by cognitive and somatic anxiety. Research on collegiate student-athletes has also shown that academic and sport commitments may be experienced as compatible or conflicting and may require coping and support suited to their combined demands (Cosh and Tully, 2015; O’Neil et al., 2021). Similar academic and performance-related expectations, however, do not carry the same psychological meaning for every student. Individual characteristics may shape whether these expectations are experienced as manageable, motivating, or stressful.

Perfectionism is one characteristic that may contribute to these differences. Contemporary models conceptualize perfectionism as multidimensional, commonly distinguishing achievement-oriented strivings from evaluative concerns and negative reactions to perceived imperfection (Stoeber and Otto, 2006; Smith et al., 2022). Meta-analyses by Smith et al. (2018) and Callaghan et al. (2024) indicate that concern-related dimensions are more consistently associated with anxiety and broader psychological distress, whereas striving-related dimensions tend to show weaker or more context-dependent associations. The Almost Perfect Scale–Revised (APS-R) operationalizes a related multidimensional structure through High Standards, Order, and Discrepancy (Slaney et al., 2001, 2002). High Standards reflects the pursuit of demanding personal goals, Order reflects a preference for organization and structure, and Discrepancy reflects the perception that one’s performance falls short of personally important standards. Research using the APS-R among undergraduates has similarly identified different patterns of association between these dimensions and anxiety, stress, and other mental health outcomes (Simon et al., 2025).

Discrepancy appears particularly relevant to the way academic and performance demands are interpreted. Its emphasis on perceived shortfalls is consistent with self-critical evaluations of one’s performance. Rassaby et al. (2021) found that greater Discrepancy among high-achieving undergraduates was associated with higher task-related and overall anxiety and poorer psychological well-being. Evidence from athlete samples points in a similar direction. Stoeber et al. (2007) found that striving for perfection was not associated with greater competitive anxiety after negative reactions to imperfection were distinguished, whereas negative reactions were consistently associated with higher anxiety. High Standards may therefore organize effort and goal pursuit without necessarily being accompanied by distress. The role of Order is less well established, particularly in relation to perceived stress and anxiety. The psychological implications of perfectionism may consequently depend not only on the standards individuals hold, but also on how they interpret and respond to perceived shortfalls.

The transactional model of stress provides a theoretical basis for understanding this appraisal process. Psychological responses depend not simply on the presence of external demands but also on how those demands are evaluated in relation to perceived coping resources (Lazarus and Folkman, 1984). Perceived stress captures the extent to which circumstances are experienced as unpredictable, uncontrollable, or overwhelming (Cohen et al., 1983). Students facing comparable demands may therefore differ markedly in the stress they report. Rice and Van Arsdale (2010) linked maladaptive perfectionism with higher perceived stress among college students, whereas Stevenson and Akram (2022) provided longitudinal evidence connecting perfectionism, self-critical thinking, and perceived stress. Perceived stress is also associated with anxiety symptoms in university populations (Beiter et al., 2015). Among Chinese college students, Wang et al. (2022) further identified perceived stress as an important correlate within the relationship between perfectionism and social anxiety. Perceived stress may therefore be relevant to understanding why discrepancy-related concerns are accompanied by greater anxiety symptoms in some students.

Evidence concerning these relationships remains limited among Chinese undergraduates in sport-related majors. Previous studies have more often examined general university students, medical students, collegiate athletes, or students in specific performance situations (Chai et al., 2020; Li et al., 2024; Kosmidou et al., 2025). Few studies have considered High Standards, Order, and Discrepancy together with perceived stress and general anxiety symptoms in a multi-site sample from this educational population. The present study therefore examined the associations of the three APS-R dimensions with perceived stress and anxiety symptoms among Chinese undergraduates in sport-related majors. We expected Discrepancy to be positively associated with perceived stress and anxiety symptoms and perceived stress to be positively associated with anxiety symptoms. Measurement analyses were used to determine whether the adapted PSS-14 should be represented by a total score or by component scores. We expected Discrepancy to be positively associated with the supported perceived-stress score or scores and with anxiety symptoms. We also expected a positive cross-sectional indirect association involving Discrepancy. Associations involving High Standards and Order were examined on an exploratory basis because previous findings for these dimensions have been less consistent.

## Materials and methods

### Study design, setting, participants

This multi-site cross-sectional study examined the associations of perfectionism dimensions with perceived stress and anxiety symptoms among full-time undergraduates enrolled in sport-related majors in mainland China. The study was reported with reference to the Strengthening the Reporting of Observational Studies in Epidemiology recommendations for cross-sectional studies (von Elm et al., 2007).

Data were collected between 1 March and 31 May 2026 from 19 universities and colleges located across seven geographical regions of mainland China: East China, South China, Central China, North China, Northeast China, Northwest China, and Southwest China. Participating institutions included sport-specialized universities or colleges and physical education schools, departments, or related academic units within comprehensive and teacher-training universities.

Participants were drawn from several sport-related majors, including Social Sports Guidance and Management, Leisure Sports, Sports Training, Wushu and Traditional National Sports, Strength and Conditioning, and Football (Soccer) Sport and Management. Eligible participants were required to: (1) be enrolled as full-time undergraduate students; (2) be studying in Years 1–4; (3) be enrolled in one of the included sport-related majors; and (4) provide electronic informed consent. Questionnaires were excluded if they were incomplete or lacked complete data for the APS-R, the contextually adapted perceived-stress measure, or the GAD-7. Only participants with complete data on all three core psychological measures were included in the analytic sample.

### Recruitment, data-collection procedure, and ethical approval

Participants were recruited through classes, academic cohorts, and, where applicable, training groups, with assistance from institutional contacts. The survey was administered anonymously through Wenjuanxing, an online questionnaire platform. Survey links or QR codes were distributed to potential participants, who voluntarily completed the questionnaire using a smartphone or computer.

Before beginning the survey, participants read an electronic information statement describing the study purpose, procedures, voluntary nature of participation, confidentiality arrangements, and their right to discontinue participation. Electronic informed consent was obtained before access to the questionnaire was granted. No personally identifying information was collected.

A total of 750 questionnaires were distributed. After 45 incomplete questionnaires or questionnaires lacking complete data on at least one core psychological measure were excluded, 705 participants were retained for analysis. The questionnaire disposition is presented in Table 1.

**Table 1:** Participant characteristics, survey disposition, and GAD-7 anxiety symptom profile.

Variable	Category	*N* (%) or M ± SD
Survey disposition	Initial questionnaires	750
	Excluded questionnaires	45 (6.0%)
	Final analytic sample	705 (94.0%)
Gender	Male	504 (71.5%)
	Female	201 (28.5%)
Age, years	Mean ± SD	19.90 ± 1.72
Only-child status	No	522 (74.0%)
	Yes	183 (26.0%)
Highest parental education	Master’s degree or above	216 (30.6%)
	Bachelor’s degree	183 (26.0%)
	Associate degree	59 (8.4%)
	Senior high/vocational secondary	212 (30.1%)
	Junior high or below	35 (5.0%)
Year of study	Year 1	330 (46.8%)
	Year 2	108 (15.3%)
	Year 3	183 (26.0%)
	Year 4	84 (11.9%)
Institution type	Sport-specialized university/college	430 (61.0%)
	Comprehensive/normal university PE department	275 (39.0%)
High-level university team member	Yes	97 (13.8%)
	No	608 (86.2%)
GAD-7 severity	Minimal, 0–4	319 (45.2%)
	Mild, 5–9	278 (39.4%)
	Moderate, 10–14	71 (10.1%)
	Severe, ≥15	37 (5.2%)
	Moderate-to-severe, ≥10	108 (15.3%)

*N* = 705. GAD-7 categories are descriptive and do not represent clinical diagnoses. The moderate-to-severe total is not an additional mutually exclusive category.

The study protocol was reviewed and approved by the Shandong Sport University Sport Science Ethics Committee on 2 December 2025 (approval no. 2025074). All procedures were conducted in accordance with the Declaration of Helsinki and the ethical principles applicable to research involving human participants.

### Sample-size considerations

During study planning, PASS 15.0 was used to examine plausible sample sizes for estimating an anxiety-symptom proportion across the prevalence range of 18.1–31.5% reported in previous research (Zhang and Jin, 2025). The planning calculations used a two-sided 95% confidence level, an absolute precision of approximately 0.05, and an allowance for incomplete questionnaires, producing a target range of approximately 246–350 participants. The final analytic sample of 705 exceeded this range and was considered adequate for the planned measurement and multivariable analyses (Wolf et al., 2013).

## Measures

### Demographic and educational characteristics

A self-administered background questionnaire collected information on gender, age, only-child status, highest parental educational attainment, year of study, institution type, and membership in a high-level university team. Institution type distinguished sport-specialized universities or colleges from physical education schools or departments within comprehensive and teacher-training universities. High-level university team membership was coded as yes or no.

### Almost perfect scale–revised (APS-R)

Perfectionism was assessed using the Chinese version of the 23-item Almost Perfect Scale–Revised (APS-R; Slaney et al., 2001; Yang et al., 2007). Items are rated on a 7-point scale ranging from 1 (*strongly disagree*) to 7 (*strongly agree*). The APS-R comprises three dimensions: High Standards (7 items), Order (4 items), and Discrepancy (12 items). Subscale scores were calculated as the mean of the items within each dimension, with higher scores indicating stronger endorsement of that dimension. In the present sample, internal consistency was acceptable for High Standards (*α* = 0.841, *ω* = 0.844), Order (*α* = 0.760, *ω* = 0.762), and Discrepancy (*α* = 0.891, *ω* = 0.892). However, none of the candidate APS-R latent models was retained, and High Standards and Order showed substantial overlap. The three scored subscales were therefore used as observed predictors in the structural analyses, and no APS-R total score was calculated. Detailed measurement results are presented in Table 2.

**Table 2:** Confirmatory measurement models, construct-level evidence, and hybrid sensitivity-model fit.

Model	*χ* ^2^	*df*	CFI	TLI	RMSEA	SRMR	Decision
APS-R one-factor model	4077.113	230	0.507	0.457	0.154 [0.150, 0.158]	0.161	Not retained
APS-R two-factor model	2091.642	229	0.761	0.736	0.107 [0.103, 0.112]	0.120	Not retained
APS-R three-correlated-factor model	2065.480	227	0.764	0.737	0.107 [0.103, 0.112]	0.118	Not retained as latent representation
PSS-14 one-factor model	2676.061	77	0.498	0.406	0.219 [0.212, 0.226]	0.242	Not retained
PSS-14 correlated two-factor model	446.565	76	0.928	0.914	0.083 [0.076, 0.091]	0.050	Retained for component scoring
PSS-14 bifactor diagnostic model	234.044	63	0.967	0.952	0.062 [0.054, 0.071]	0.043	General factor not retained for total scoring
GAD-7 one-factor model	80.899	14	0.981	0.971	0.082 [0.065, 0.100]	0.022	Retained
Hybrid SEM sensitivity model	842.546	246	0.941	0.933	0.059 [0.054, 0.063]	0.044	Retained as sensitivity model

Construct	Loading range	α	CR	AVE	Key evidence	Interpretation
Panel B. construct-level evidence						
High Standards	0.568–0.726	0.841	0.844	0.439	HTMT with Order = 0.951	Convergent validity limited; not distinct from Order
Order	0.577–0.726	0.760	0.762	0.447	HTMT with High Standards = 0.951	Convergent validity limited; not distinct from High standards
Discrepancy	0.287–0.761	0.891	0.893	0.417	HTMT with High standards/order = 0.460/0.323	Reliability acceptable; convergent validity limited
PSS low-coping component	0.598–0.871	0.896	0.898	0.561	HTMT with negative-stress component = 0.091	Supported
PSS negative-stress component	0.651–0.799	0.888	0.889	0.534	HTMT with low-coping component = 0.091	Supported
Anxiety symptoms	0.783–0.842	0.931	0.931	0.660	Single-factor construct	Supported

Diagnostic	Estimate	Interpretation
Panel C. PSS-14 total-score diagnostics		
Correlation between observed component scores	*r* = −0.036, *p* = 0.339	The two score clusters were not positively integrated
Latent correlation in the correlated two-factor model	*r* = −0.020, *p* = 0.637	No evidence of a common higher-order direction
Bifactor explained common variance	ECV = 0.086	General factor accounted for little common variance
Bifactor reliability	ωH = 0.062; ω total = 0.905	High total reliability was driven mainly by specific factors
Percentage of uncontaminated correlations	PUC = 0.538	Considered jointly with ECV and ωH; total score not retained

Models were estimated by maximum likelihood with item responses treated as approximately continuous. The APS-R models were specified without data-driven residual correlations. CR, composite reliability; AVE, average variance extracted; HTMT, heterotrait–monotrait ratio; ECV, explained common variance; ωH, omega hierarchical; PUC, percentage of uncontaminated correlations. The PSS-14 bifactor model was used to assess whether a defensible general score existed; model fit alone was not treated as evidence for total-score interpretation.

### Contextually adapted perceived-stress measure based on the Chinese PSS-14

Perceived stress was assessed using a 14-item measure contextually adapted from the original Perceived Stress Scale (PSS; Cohen et al., 1983) and the Chinese version reported by Yang and Huang (2003). The PSS assesses the extent to which individuals appraise their circumstances as unpredictable, uncontrollable, or overwhelming. Previous research has examined the reliability and factorial structure of the Chinese PSS-14, while also indicating that item performance and dimensional structure may vary across samples (Huang et al., 2020). All items in the present study referred to experiences during the previous month.

Of the 14 items, eight retained the substantive wording of the Chinese PSS-14, four were contextually modified, and two underwent minor linguistic adjustments without changing their intended meaning. Items 1, 8, 11, and 12 were modified to represent training–coursework conflict, imbalance between training load and recovery, pressure associated with physical testing, selection or competition outcomes, and perceived loss of control when competition periods overlapped with examinations. The study team completed the contextual adaptation between 15 October and 25 November 2025 during questionnaire preparation. Selected items were revised with reference to the corresponding Chinese PSS-14 items and the academic and sport-related demands relevant to the study population. The source wording, wording administered in the present study, and nature of each modification are presented in Supplementary Table S1.

Items were rated on a five-point scale ranging from 0 (*never*) to 4 (*very often*). Items 4, 5, 6, 7, 9, 10, and 13 were reverse scored. A conventional total score could range from 0 to 56, but its interpretability was evaluated before the structural analyses. The correlated two-factor model fitted substantially better than the one-factor model, whereas the bifactor diagnostics indicated that the general factor was too weak to support interpretation of the total score as a general perceived-stress measure. Two component scores were therefore calculated and analyzed separately: low perceived coping, comprising the seven reverse-scored positively worded items, and negative stress appraisal, comprising Items 1, 2, 3, 8, 11, 12, and 14. Each component ranged from 0 to 28, with higher scores indicating lower perceived coping or more negative stress appraisal, respectively. Internal consistency was good for both low perceived coping (*α* = 0.896, *ω* = 0.898) and negative stress appraisal (*α* = 0.888, *ω* = 0.889). Detailed measurement results are presented in Table 2.

### Generalized anxiety disorder-7 (GAD-7)

Anxiety symptoms during the previous 2 weeks were assessed using the seven-item Generalized Anxiety Disorder scale (GAD-7; Spitzer et al., 2006). Items are rated from 0 (*not at all*) to 3 (*nearly every day*), yielding a possible total score of 0–21. For descriptive purposes, scores were categorized as minimal (0–4), mild (5–9), moderate (10–14), or severe (15–21). A score of 10 or higher was used to describe moderate-to-severe anxiety symptoms. These categories summarized symptom severity and were not treated as clinical diagnoses. In the present sample, the GAD-7 showed excellent internal consistency (*α* = 0.931, *ω* = 0.931), and the one-factor model was retained. Detailed measurement results are presented in Table 2.

### Statistical analysis

Analyses were conducted in Python 3.13.5 using Statsmodels 0.14.6 and Semopy, with pandas 2.2.3, NumPy 2.3.5, SciPy 1.17.0, and Pyreadstat 1.3.5 used for data management and supporting calculations. Participant characteristics and GAD-7 severity categories were summarized using means and standard deviations or frequencies and percentages, as appropriate. Questionnaires with incomplete APS-R, PSS-14, or GAD-7 data were excluded. Item coding and response ranges were checked before analysis. Two responses to GAD-7 Item 6 exceeded the valid range and were recoded to the maximum valid score of 3; this correction did not alter the corresponding symptom-severity classifications. Confirmatory factor analyses and the hybrid structural equation model were estimated using maximum likelihood, with item responses treated as approximately continuous.

For the APS-R, one-factor, two-factor, and three-correlated-factor models were compared. In the two-factor alternative, High Standards and Order were represented by a common factor, whereas Discrepancy was modeled separately. For the contextually adapted PSS-14, one-factor, correlated two-factor, and bifactor models were evaluated. The correlated two-factor model separated the seven reverse-scored positive items from the seven negative or contextually framed items. The bifactor model included a general factor and two orthogonal specific factors. The GAD-7 was evaluated using a one-factor model. Model fit was assessed using *χ*^2^, the comparative fit index, Tucker–Lewis index, root mean square error of approximation with its 90% confidence interval, and standardized root mean square residual (Hu and Bentler, 1999). Explained common variance, percentage of uncontaminated correlations, omega hierarchical, and omega total were used to assess whether the bifactor general factor supported interpretation of a PSS-14 total score (Rodriguez et al., 2016). Internal consistency was evaluated using Cronbach’s alpha and McDonald’s omega. Composite reliability, average variance extracted, latent-factor correlations, and the heterotrait–monotrait ratio were examined as indicators of convergent and discriminant validity (Fornell and Larcker, 1981; Henseler et al., 2015). Configural, metric, and scalar invariance of the PSS-14 two-factor and GAD-7 one-factor models was examined across gender and institution type, with changes in CFI and RMSEA used to evaluate increasingly constrained models (Chen, 2007).

The primary observed-score path analysis used High Standards, Order, and Discrepancy scores as predictors; low perceived coping and negative stress appraisal as parallel intervening variables; and the GAD-7 total score as the anxiety-symptom outcome. Each perceived-stress component was regressed on the three APS-R scores. Anxiety symptoms were regressed on both perceived-stress components and the three APS-R scores. Unstandardized and standardized coefficients, HC3 heteroskedasticity-robust standard errors, 95% confidence intervals, and explained variance were reported. Component-specific and total parallel indirect associations were estimated using 5,000 percentile-bootstrap resamples (Preacher and Hayes, 2008). An indirect association was considered statistically distinguishable from zero when its 95% bootstrap confidence interval excluded zero. Given the cross-sectional design, the estimates were interpreted as statistical associations rather than temporal or causal mediation (Maxwell and Cole, 2007; Maxwell et al., 2011).

The primary model was estimated without covariates. An expanded sensitivity analysis adjusted for gender, age, year of study, institution type, high-level university team membership, only-child status, and parental education and used HC3 heteroskedasticity-robust standard errors. Variance-inflation factors were examined for the adjusted model. A hybrid SEM sensitivity model retained the three APS-R scores as observed predictors and represented the two PSS-14 components and anxiety symptoms as latent factors. A single-factor CFA was fitted as a common-method diagnostic (Podsakoff et al., 2003), and an age sensitivity analysis excluded the single participant aged 39 years. The de-identified analytic dataset retained institution type but did not contain verified participant-level identifiers for the 19 individual universities; university-level intraclass correlations and institution-clustered estimates were therefore not calculated.

## Results

### Participant characteristics

A total of 750 questionnaires were recorded. After 45 incomplete questionnaires were excluded, 705 participants were included in the analytic sample, corresponding to a usable questionnaire rate of 94.0%. The sample comprised 504 men (71.5%) and 201 women (28.5%), with a mean age of 19.90 years (SD = 1.72).

Based on the GAD-7 severity categories, 319 participants (45.2%) were classified in the minimal range, 278 (39.4%) in the mild range, 71 (10.1%) in the moderate range, and 37 (5.2%) in the severe range. In total, 108 participants (15.3%) scored 10 or above, indicating moderate-to-severe anxiety symptoms. Additional demographic and educational characteristics are presented in Table 1.

### Measurement summary, descriptive statistics, and bivariate correlations

Sample-specific psychometric findings and the resulting scoring decisions are summarized in the corresponding measure subsections, with complete model-fit and construct-level results reported in Table 2. None of the candidate APS-R latent models was retained; the two-component representation of the adapted PSS-14 and the one-factor GAD-7 model were retained for the subsequent analyses. Descriptive statistics, internal-consistency estimates, and the full bivariate correlation matrix are presented in Table 3.

**Table 3:** Descriptive statistics, internal consistency, and bivariate correlations.

Variable	Items	*M*	*SD*	α	ω
1. High standards	7	5.17	0.87	0.841	0.844
2. Order	4	5.38	0.90	0.760	0.762
3. Discrepancy	12	4.19	0.97	0.891	0.892
4. Low perceived coping	7	13.67	5.67	0.896	0.898
5. Negative stress appraisal	7	9.79	5.47	0.888	0.889
6. Anxiety symptoms	7	5.49	4.63	0.931	0.931

Variable	1	2	3	4	5	6
Panel B. pearson correlations						
1. High standards	—	0.763***	0.363***	−0.144***	−0.052	−0.065
2. Order		—	0.228***	−0.187***	−0.120**	−0.118**
3. Discrepancy			—	0.242***	0.247***	0.298***
4. Low perceived coping				—	−0.036	0.132***
5. Negative stress appraisal					—	0.616***
6. Anxiety symptoms						—

*N*, 705. APS-R subscales are item-mean scores. The two perceived-stress component scores and anxiety symptoms are item-sum scores. Higher low-perceived-coping scores indicate lower perceived coping; higher negative-stress-appraisal scores indicate more negative stress appraisal. *p < 0.05; **p < 0.01; ***p < 0.001.

High Standards was negatively correlated with low perceived coping (*r* = −0.144, *p* < 0.001) but was not significantly correlated with negative stress appraisal or anxiety symptoms. Order was negatively correlated with low perceived coping (*r* = −0.187, *p* < 0.001), negative stress appraisal (*r* = −0.120, *p* < 0.01), and anxiety symptoms (*r* = −0.118, *p* < 0.01). Discrepancy was positively correlated with low perceived coping (*r* = 0.242, *p* < 0.001), negative stress appraisal (*r* = 0.247, *p* < 0.001), and anxiety symptoms (*r* = 0.298, *p* < 0.001). Low perceived coping and negative stress appraisal were not significantly correlated with each other (*r* = −0.036, *p* = 0.339). Both components were positively correlated with anxiety symptoms, although the association was substantially stronger for negative stress appraisal (*r* = 0.616) than for low perceived coping (*r* = 0.132; both *p* < 0.001).

### Primary observed-score associations

**Figure 1:**
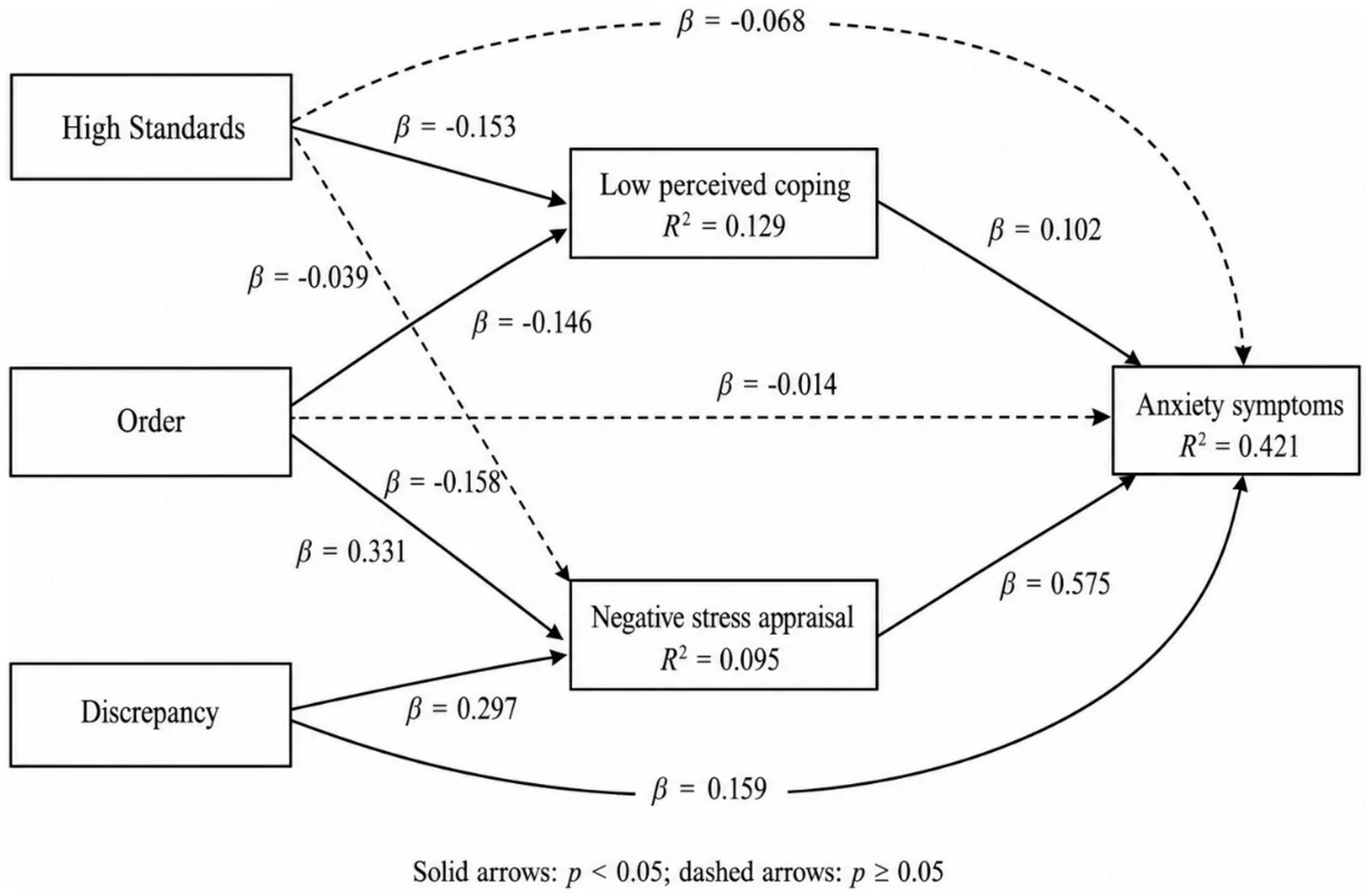
Dimension-specific structural model linking APS-R dimensions, perceived-stress components, and anxiety symptoms. Note. Values are standardized regression coefficients (*β*) from the unadjusted observed-score model. Solid arrows indicate *p* < 0.05, whereas dashed arrows indicate nonsignificant direct paths. *R*^2^ values indicate the variance explained in each endogenous score. Higher low-perceived-coping scores indicate lower perceived coping. Covariances among the three APS-R scores are omitted for clarity.

The primary path model explained 12.9% of the variance in low perceived coping, 9.5% of the variance in negative stress appraisal, and 42.1% of the variance in anxiety symptoms. Discrepancy was positively associated with low perceived coping (*β* = 0.331, *p* < 0.001), negative stress appraisal (*β* = 0.297, *p* < 0.001), and anxiety symptoms (*β* = 0.159, *p* < 0.001). Order was negatively associated with both low perceived coping (*β* = −0.146, *p* = 0.009) and negative stress appraisal (*β* = −0.158, *p* = 0.012) but was not directly associated with anxiety symptoms (*β* = −0.014, *p* = 0.751). High Standards was negatively associated with low perceived coping (*β* = −0.153, *p* = 0.014) but not with negative stress appraisal (*β* = −0.039, *p* = 0.556) or anxiety symptoms (*β* = −0.068, *p* = 0.169). Low perceived coping (*β* = 0.102, *p* = 0.001) and negative stress appraisal (*β* = 0.575, *p* < 0.001) were independently associated with anxiety symptoms. Standardized path coefficients are shown in Figure 1, and complete estimates are reported in Table 4.

**Table 4:** Primary observed-score path coefficients and bootstrap indirect associations.

Path	B	HC3 SE	*β*	*p*	95% CI for B	Outcome *R*^2^
Panel A. direct associations						
High standards → low perceived coping	−0.992	0.402	−0.153	0.014	[−1.781, −0.204]	0.129
Order → low perceived coping	−0.917	0.353	−0.146	0.009	[−1.610, −0.224]	0.129
Discrepancy → low perceived coping	1.937	0.259	0.331	< 0.001	[1.428, 2.445]	0.129
High standards → negative stress appraisal	−0.246	0.418	−0.039	0.556	[−1.066, 0.573]	0.095
Order → negative stress appraisal	−0.960	0.381	−0.158	0.012	[−1.707, −0.214]	0.095
Discrepancy → negative stress appraisal	1.680	0.256	0.297	< 0.001	[1.178, 2.183]	0.095
Low perceived coping → anxiety symptoms	0.083	0.025	0.102	0.001	[0.033, 0.133]	0.421
Negative stress appraisal → anxiety symptoms	0.486	0.028	0.575	< 0.001	[0.432, 0.541]	0.421
High standards → anxiety symptoms	−0.360	0.262	−0.068	0.169	[−0.872, 0.153]	0.421
Order → anxiety symptoms	−0.073	0.231	−0.014	0.751	[−0.527, 0.380]	0.421
Discrepancy → anxiety symptoms	0.760	0.189	0.159	< 0.001	[0.389, 1.131]	0.421

Indirect association	Effect	95% bootstrap CI	Standardized effect	95% CI for standardized effect
Panel B. bootstrap indirect associations				
High standards: via low perceived coping	−0.082	[−0.168, −0.014]	−0.016	[−0.032, −0.003]
High standards: via negative stress appraisal	−0.120	[−0.507, 0.264]	−0.023	[−0.096, 0.050]
High standards: total across both components	−0.202	[−0.587, 0.190]	−0.038	[−0.112, 0.036]
Order: via low perceived coping	−0.076	[−0.168, −0.012]	−0.015	[−0.033, −0.002]
Order: via negative stress appraisal	−0.467	[−0.830, −0.110]	−0.091	[−0.160, −0.021]
Order: total across both components	−0.543	[−0.918, −0.179]	−0.106	[−0.178, −0.035]
Discrepancy: via low perceived coping	0.161	[0.065, 0.261]	0.034	[0.014, 0.055]
Discrepancy: via negative stress appraisal	0.817	[0.565, 1.078]	0.171	[0.119, 0.223]
Discrepancy: total across both components	0.978	[0.721, 1.243]	0.205	[0.152, 0.260]

B, unstandardized coefficient; *β*, standardized coefficient; HC3 SE, heteroskedasticity-robust standard error. Direct-path confidence intervals use HC3 standard errors. Indirect associations were estimated using 5,000 percentile-bootstrap resamples. The two perceived-stress components were entered in parallel. A bootstrap interval excluding zero was treated as statistically distinguishable from zero. The estimates are cross-sectional statistical associations and do not establish temporal mediation.

For Discrepancy, the standardized indirect association was 0.034 through low perceived coping, 95% bootstrap CI [0.014, 0.055], and 0.171 through negative stress appraisal, 95% CI [0.119, 0.223]. Its total indirect association across the two components was 0.205, 95% CI [0.152, 0.260]. The total indirect association for Order was −0.106, 95% CI [−0.178, −0.035], with statistically distinguishable component-specific associations through low perceived coping and negative stress appraisal. High Standards had a small indirect association through low perceived coping [−0.016, 95% CI (−0.032, −0.003)], but its indirect association through negative stress appraisal and its total indirect association across both components were not statistically distinguishable from zero [total = −0.038, 95% CI (−0.112, 0.036)].

### Sensitivity and robustness analyses

After adjustment for gender, age, year of study, institution type, high-level university team membership, only-child status, and parental education, the focal pattern remained stable. Discrepancy remained positively associated with both perceived-stress components and anxiety symptoms. Order remained negatively associated with both perceived-stress components, and High Standards remained negatively associated only with low perceived coping. The total adjusted indirect associations remained statistically distinguishable from zero for Discrepancy [0.156, 95% bootstrap CI (0.108, 0.204)] and Order [−0.073, 95% CI (−0.131, −0.018)], but not for High Standards [−0.024, 95% CI (−0.081, 0.040)]. The adjusted model explained 50.8% of the variance in anxiety symptoms, and the maximum nonconstant variance-inflation factor was 2.74.

The hybrid SEM sensitivity model yielded *χ*^2^(246) = 842.546, CFI = 0.941, TLI = 0.933, RMSEA = 0.059, 90% CI [0.054, 0.063], and SRMR = 0.044. Its structural estimates supported the same interpretation as the primary model. Scalar invariance was supported for the PSS-14 two-factor and GAD-7 one-factor models across gender and institution type. The single-factor CFA yielded *χ*^2^(902) = 12790.109, CFI = 0.324, TLI = 0.291, RMSEA = 0.137, 90% CI [0.135, 0.139], and SRMR = 0.188. Excluding the participant aged 39 years did not alter the focal directions or significance pattern. Complete sensitivity and robustness results are presented in Supplementary Table S2.

## Discussion

The three APS-R subscales showed distinct observed-score associations with perceived stress and anxiety symptoms. Discrepancy displayed the clearest adverse pattern, whereas High Standards and Order were associated mainly with lower perceived-stress scores rather than directly with anxiety symptoms. Negative stress appraisal was more strongly associated with anxiety symptoms than low perceived coping, and the main pattern was unchanged in the supplementary analyses.

Discrepancy showed the clearest adverse pattern. Slaney et al. (2001) conceptualized this APS-R dimension as the perceived gap between personal standards and actual performance. A recurring sense of falling short may involve dissatisfaction and self-critical evaluation (Rice et al., 2019), particularly in settings characterized by repeated assessment, visible performance criteria, and feedback on academic or practical competence. Among high-achieving undergraduates, Rassaby et al. (2021) found that higher Discrepancy was associated with greater task-related and general anxiety and poorer psychological well-being. This accords with broader evidence that perfectionistic concerns are more strongly associated with anxiety and psychological distress than perfectionistic strivings (Callaghan et al., 2024). In sport, negative reactions to imperfection have shown more consistent associations with competitive anxiety than striving for perfection itself (Stoeber et al., 2007). Hill et al. (2018) similarly found that perfectionistic concerns were broadly associated with maladaptive motivational, emotional, and well-being outcomes in athletes, whereas perfectionistic strivings showed a more mixed pattern. Together, these findings suggest that how perceived shortfalls are evaluated may be more relevant to distress than the level of personal standards alone.

The absence of a direct association between High Standards and anxiety symptoms accords with multidimensional accounts in which achievement-oriented striving is not inherently maladaptive; its implications depend on rigidity, evaluative concern, and responses to setbacks (Stoeber and Otto, 2006). Among physical education and sport students completing practical examinations, Kosmidou et al. (2025) found that High Standards was associated with greater self-confidence rather than somatic or cognitive anxiety, whereas Discrepancy was more closely related to anxiety in the rhythmic-gymnastics examination condition. Because that study examined situation-specific state anxiety in a Greek sample, it provides contextually relevant rather than directly confirmatory evidence. The present pattern nevertheless supports distinguishing ambitious standards from persistent dissatisfaction with one’s performance. Given the substantial overlap between High Standards and Order, the High Standards coefficient is best interpreted as a conditional association rather than evidence of a general benefit of demanding goals.

Order’s association with lower perceived-stress scores may reflect greater predictability when students coordinate multiple academic and practice-based requirements. Sport-related undergraduate education in China encompasses several distinct majors whose curricula should not be treated as uniform (Ministry of Education of the People’s Republic of China, 2025a, 2025b). Current physical education training plans nevertheless illustrate how theoretical coursework may be combined with sport-skill assessment, teaching-skill examinations, field-based education, and training or competition-related practice (Beijing Sport University, 2024; Capital University of Physical Education and Sports, 2021). Student-athlete research offers an imperfect but relevant comparison: schedule clashes, fatigue, and time-management difficulties have been identified when higher education and intensive sport commitments must be coordinated (Cosh and Tully, 2015; Lopes Dos Santos et al., 2020), and academic and sport commitments may be experienced as either compatible or conflicting (O’Neil et al., 2021). Organization and routine could therefore coincide with less negative appraisal of competing demands. Order does not, however, directly measure planning or time-management ability, and the study did not assess self-regulation, practicum exposure, or access to support. This contextual interpretation remains provisional. High Standards and Order were also not clearly differentiated in the measurement analyses, so their coefficients are best understood as conditional associations among closely related APS-R scores rather than as effects of clearly separable traits.

The contrast between the two perceived-stress components was one of the clearest features of the model. Negative stress appraisal had a substantially stronger association with anxiety symptoms than low perceived coping, and the estimated indirect associations involving Order and Discrepancy were correspondingly larger through negative stress appraisal. A similar bivariate asymmetry has been reported among Chinese adolescents, for whom the negatively worded perceived-helplessness component showed a stronger association with anxiety than the positively worded perceived-self-efficacy component (Liu et al., 2020). The stronger coefficient may reflect a closer substantive relationship between anxiety and experiences of uncontrollability or overload, but overlap in negative wording and emotional content may also contribute to the association. At the measurement level, the near-zero correlation between the two component scores and the weak bifactor general factor did not support interpreting the PSS-14 total score as a sufficiently coherent general-stress indicator in this sample. Huang et al. (2020) similarly reported weak or nonsignificant associations between positive and negative-item PSS factors in a large Chinese community sample. The meaning of the present two-component structure remains unresolved: it may reflect a substantive distinction between negative appraisal and perceived coping, while experimental evidence indicates that opposing item wording can also contribute to apparent multidimensionality and measurement error (Pedersen et al., 2024). Because item content and wording direction were fully aligned, and six items underwent contextual or linguistic modification, the two scores are best regarded as provisional components for the present sample rather than established PSS-14 subscales. The APS-R latent models likewise did not support three clearly differentiated constructs, particularly for High Standards and Order. The primary findings should therefore be interpreted at the scored-subscale level. Within this boundary, the main observed-score pattern remained unchanged across the expanded covariate, hybrid SEM, and age-sensitivity analyses.

Recent qualitative work by Chen and Ni (2024) indicates that Chinese students with sport-related identities may encounter education and culture-specific stereotypes, reinforcing the need to interpret these findings within their educational context. In programs that combine academic work, practical assessment, field placement, and sport-related performance demands, the distinction between ambitious standards and persistent perceptions of falling short may be particularly relevant. The findings point toward supporting flexible self-evaluation and manageable stress appraisal rather than focusing on the level of personal standards alone. Process-focused feedback, flexible goal adjustment, and coordination among academic, training, practicum, and student-support services warrant evaluation in future intervention research.

Theoretically, the present findings support distinguishing demanding personal standards from evaluative discrepancy when examining perfectionism-related distress. At the observed-score level, the clearest adverse pattern was associated with persistent perceptions of falling short rather than with High Standards alone. The findings also add nuance to appraisal-based interpretations of stress by indicating that negative appraisal of demands was more closely aligned with anxiety symptoms than low perceived coping. Taken together, these patterns suggest that the psychological meaning assigned to perceived shortfalls and demands may be more consequential than the level of aspiration itself. From a practical perspective, assessment and student support should therefore avoid treating demanding standards as inherently problematic and instead give greater attention to rigid self-evaluation, repeated dissatisfaction despite adequate performance, and perceptions that academic or performance demands are uncontrollable. Recent qualitative work indicates that Chinese students with sport-related identities may encounter education and culture-specific pressures and stereotypes, underscoring the importance of context-sensitive support (Chen and Ni, 2024). In programs combining academic coursework, practical assessment, field placement, and sport-related performance demands, process-focused feedback, flexible goal adjustment, and coordination among academic, training, practicum, and student-support services may help students maintain ambitious but adaptable goals. These theoretical and practical interpretations remain provisional because High Standards and Order were not clearly differentiated, the two adapted PSS-14 components were aligned with item wording, and the proposed applications have yet to be evaluated in longitudinal and intervention research.

The study has several strengths, including a geographically dispersed multi-site sample and the examination of all three APS-R subscales within the same analytical framework. Its conclusions remain subject to several limitations. Because perfectionism, perceived stress, and anxiety symptoms were measured concurrently, the model-estimated indirect associations represent cross-sectional statistical decompositions rather than temporal mediation. Reciprocal pathways remain plausible; anxiety symptoms may heighten perceptions of uncontrollability, and perfectionism-related concerns and perceived stress may share unmeasured antecedents. Longitudinal and repeated-measures studies are needed to establish temporal ordering and to examine whether the associations vary across academic, training, practicum, and competition periods. All principal variables were assessed by self-report, and the poorer fit of the single-factor CFA does not rule out shared response tendencies or other common-method influences. The measurement limitations of the APS-R and adapted PSS-14 also restrict construct-level interpretation. Future work should combine cognitive interviewing, test–retest assessment, and external validation using an unmodified Chinese version. Detailed information on training load, practicum exposure, sleep and recovery, recent injury, fear of failure, coping, emotion regulation, and access to psychological support was unavailable, leaving open the possibility of residual confounding. Although participants were recruited from 19 institutions, verified participant-level university identifiers were not retained in the de-identified analytic dataset, precluding direct estimation and correction of institution-level clustering; adjustment for institution type does not substitute for university-level clustering correction. Men represented 71.5% of the sample. Scalar invariance of the PSS-14 and GAD-7 supports comparability of these measures across gender, but it does not resolve the sample imbalance or establish APS-R invariance. Finally, the absence of a comparison group from other academic disciplines prevents conclusions about whether the observed pattern is specific to sport-related majors. Generalization to professional athletes, students in other fields, or populations outside China should therefore remain cautious.

## Conclusion

Among Chinese undergraduates in sport-related majors, the psychologically adverse aspect of perfectionism appears to lie primarily in persistent perceptions of falling short of personally important standards rather than in demanding standards themselves. Negative appraisal of academic and performance demands was more closely aligned with anxiety symptoms than perceived difficulties in coping, highlighting the importance of how students interpret pressure and setbacks. These findings support directing attention toward rigid self-evaluation, repeated dissatisfaction, and perceived uncontrollability while preserving ambitious but flexible goals. The implications remain provisional because the APS-R dimensions were not fully distinct, the adapted PSS-14 components were aligned with item wording, and the cross-sectional design cannot establish temporal or causal pathways.

## Data Availability

The datasets presented in this article are not readily available because the de-identified data supporting the findings of this study are available from the corresponding author upon reasonable request, subject to applicable ethical and institutional requirements. Requests to access the datasets should be directed to XX, xxq152@gmail.com.
