# Supplementary material for: Associations of perfectionism dimensions with perceived stress and anxiety symptoms among Chinese undergraduates in sport-related majors: a multi-site cross-sectional study
Source: Front Psychol. 2026 Aug 17;17:1910894. doi: 10.3389/fpsyg.2026.1910894 (PMC13526677; doi:10.3389/fpsyg.2026.1910894)
Supplement: Supplementary file 1 [file Table_1.docx]

| **Supplementary Table S1.** Item-level record of the perceived-stress measure contextually adapted from the Chinese PSS-14 | | | | |
| --- | --- | --- | --- | --- |
| **Item** | **Original Chinese PSS-14 wording**  **(English translation)** | **Wording administered in this study**  **(English translation)** | **Nature and purpose of adaptation** | **Reverse scored** |
| 1 | In the last month, how often have you been upset because of something that happened unexpectedly? | In the last month, how often have you felt it was difficult to meet both training and coursework demands when their schedules conflicted? | Contextual replacement to represent conflict between training and coursework. | No |
| 2 | In the last month, how often have you felt that you were unable to control the important things in your life? | In the last month, how often have you felt that you were unable to control the important things in your life? | No change. | No |
| 3 | In the last month, how often have you felt nervous and stressed? | In the last month, how often have you felt nervous and stressed? | No change. | No |
| 4 | In the last month, how often have you successfully dealt with irritating life hassles? | In the last month, how often have you successfully dealt with irritating life hassles? | No change. | Yes |
| 5 | In the last month, how often have you felt that you were effectively coping with important changes occurring in your life? | In the last month, how often have you felt that you could effectively handle important changes happening in your life? | Minor linguistic adjustment only; the substantive meaning was retained. | Yes |
| 6 | In the last month, how often have you felt confident about your ability to handle your personal problems? | In the last month, how often have you felt confident about your ability to handle your personal problems? | No change. | Yes |
| 7 | In the last month, how often have you felt that things were going your way? | In the last month, how often have you felt that things were going your way? | No change. | Yes |
| 8 | In the last month, how often have you found that you could not cope with all the things that you had to do? | In the last month, how often have you felt mentally and physically unable to cope when your training load and recovery were imbalanced? | Contextual narrowing to represent difficulty coping with an imbalance between training load and recovery. | No |
| 9 | In the last month, how often have you been able to control irritations in your life? | In the last month, how often have you been able to control irritations in your life? | No change. | Yes |
| 10 | In the last month, how often have you felt that you were on top of things? | In the last month, how often have you felt that you were on top of things? | No change. | Yes |
| 11 | In the last month, how often have you been angered because of things that happened that were outside your control? | In the last month, how often have you felt a persistent psychological burden due to pressure from fitness tests, selection, or competition results? | Contextual replacement to represent pressure arising from fitness tests, selection, and competition outcomes. | No |
| 12 | In the last month, how often have you found yourself thinking about things that you have to accomplish? | In the last month, how often have you felt that your schedule and rhythm were out of control when the competition season overlapped with examination weeks? | Contextual replacement to represent perceived loss of control when competition and examination periods overlap. | No |
| 13 | In the last month, how often have you been able to control the way you spend your time? | In the last month, how often have you been able to manage the way you schedule your time? | Minor linguistic adjustment only; the substantive meaning was retained. | Yes |
| 14 | In the last month, how often have you felt that difficulties were piling up so high that you could not overcome them? | In the last month, how often have you felt that difficulties were piling up so high that you could not overcome them? | No change. | No |
| Note. PSS-14 = 14-item Perceived Stress Scale. The wording in the second and third columns is presented in English for publication; the questionnaire was administered in Chinese. Items 1, 8, 11, and 12 were contextually modified. Items 1, 11, and 12 involved contextual replacement, whereas Item 8 narrowed a general overload item to training-load/recovery imbalance. Items 5 and 13 contained minor linguistic adjustments without substantive change. Items 4, 5, 6, 7, 9, 10, and 13 were reverse scored. All items referred to experiences during the previous month and were rated from 0 (never) to 4 (very often). | | | | |
