# Supplementary material for: Associations of perfectionism dimensions with perceived stress and anxiety symptoms among Chinese undergraduates in sport-related majors: a multi-site cross-sectional study
Source: Front Psychol. 2026 Aug 17;17:1910894. doi: 10.3389/fpsyg.2026.1910894 (PMC13526677; doi:10.3389/fpsyg.2026.1910894)
Supplement: Supplementary file 2 [file Table_2.docx]

| **Supplementary Table S2. Measurement-invariance, covariate-adjusted, and robustness analyses** | | | | | | | | | | | | | | | | | | | | |
| --- | --- | --- | --- | --- | --- | --- | --- | --- | --- | --- | --- | --- | --- | --- | --- | --- | --- | --- | --- | --- |
| **Panel A. Covariate-adjusted observed-score model** | | | | | | | | | | | | | | | | | | | | |
| **Path** | | | | | | | | | | **Primary *β*** | | **Adjusted *β*** | | | **95% robust CI for adjusted *β*** | | | | | **Adjusted *p*** |
| High Standards → Low perceived coping | | | | | | | | | | −0.153 | | −0.153 | | | [−0.277, −0.028] | | | | | 0.016 |
| Order → Low perceived coping | | | | | | | | | | −0.146 | | −0.134 | | | [−0.246, −0.022] | | | | | 0.019 |
| Discrepancy → Low perceived coping | | | | | | | | | | 0.331 | | 0.317 | | | [0.228, 0.405] | | | | | < 0.001 |
| High Standards → Negative stress appraisal | | | | | | | | | | −0.039 | | −0.028 | | | [−0.154, 0.098] | | | | | 0.662 |
| Order → Negative stress appraisal | | | | | | | | | | −0.158 | | −0.131 | | | [−0.247, −0.014] | | | | | 0.028 |
| Discrepancy → Negative stress appraisal | | | | | | | | | | 0.297 | | 0.276 | | | [0.189, 0.363] | | | | | < 0.001 |
| Low perceived coping → Anxiety symptoms | | | | | | | | | | 0.102 | | 0.067 | | | [0.010, 0.123] | | | | | 0.021 |
| Negative stress appraisal → Anxiety symptoms | | | | | | | | | | 0.575 | | 0.487 | | | [0.419, 0.555] | | | | | < 0.001 |
| High Standards → Anxiety symptoms | | | | | | | | | | −0.068 | | −0.073 | | | [−0.163, 0.017] | | | | | 0.111 |
| Order → Anxiety symptoms | | | | | | | | | | −0.014 | | 0.001 | | | [−0.085, 0.086] | | | | | 0.989 |
| Discrepancy → Anxiety symptoms | | | | | | | | | | 0.159 | | 0.160 | | | [0.089, 0.231] | | | | | < 0.001 |
| **Outcome** | | | | | **Primary R²** | | | | | | | | | | **Adjusted R²** | | | | | |
| Low perceived coping | | | | | 0.129 | | | | | | | | | | 0.146 | | | | | |
| Negative stress appraisal | | | | | 0.095 | | | | | | | | | | 0.188 | | | | | |
| Anxiety symptoms | | | | | 0.421 | | | | | | | | | | 0.508 | | | | | |
| **Panel B. Adjusted bootstrap indirect associations** | | | | | | | | | | | | | | | | | | | | |
| **Indirect association** | | | | | | **Primary standardized effect** | | | | | **Primary 95% CI** | | | | **Adjusted standardized effect** | | | | **Adjusted 95% CI** | |
| High Standards: via low perceived coping | | | | | | −0.016 | | | | | [−0.032, −0.003] | | | | −0.010 | | | | [−0.024, −0.000] | |
| High Standards: via negative stress appraisal | | | | | | −0.023 | | | | | [−0.096, 0.050] | | | | −0.014 | | | | [−0.070, 0.049] | |
| High Standards: total parallel indirect | | | | | | −0.038 | | | | | [−0.112, 0.036] | | | | −0.024 | | | | [−0.081, 0.040] | |
| Order: via low perceived coping | | | | | | −0.015 | | | | | [−0.033, −0.002] | | | | −0.009 | | | | [−0.022, −0.000] | |
| Order: via negative stress appraisal | | | | | | −0.091 | | | | | [−0.160, −0.021] | | | | −0.064 | | | | [−0.121, −0.009] | |
| Order: total parallel indirect | | | | | | −0.106 | | | | | [−0.178, −0.035] | | | | −0.073 | | | | [−0.131, −0.018] | |
| Discrepancy: via low perceived coping | | | | | | 0.034 | | | | | [0.014, 0.055] | | | | 0.021 | | | | [0.003, 0.041] | |
| Discrepancy: via negative stress appraisal | | | | | | 0.171 | | | | | [0.119, 0.223] | | | | 0.134 | | | | [0.090, 0.183] | |
| Discrepancy: total parallel indirect | | | | | | 0.205 | | | | | [0.152, 0.260] | | | | 0.156 | | | | [0.108, 0.204] | |
| Note. The adjusted model included gender, age, year of study, institution type, high-level university team membership, only-child status, and parental education. Parental education was represented by indicator variables with senior high/vocational secondary education as the reference category. HC3 standard errors were used. The maximum nonconstant VIF was 2.74. | | | | | | | | | | | | | | | | | | | | |
| **Panel C. Hybrid SEM sensitivity model** | | | | | | | | | | | | | | | | | | | | |
| **Structural path** | | | | | | | | **Standardized β** | | | | | | | | | ***p*** | | | |
| High Standards → Low | | | | | | | | −0.164 | | | | | | | | | 0.006 | | | |
| Order → Low | | | | | | | | −0.144 | | | | | | | | | 0.012 | | | |
| Discrepancy → Low | | | | | | | | 0.338 | | | | | | | | | < 0.001 | | | |
| High Standards → Neg | | | | | | | | −0.048 | | | | | | | | | 0.429 | | | |
| Order → Neg | | | | | | | | −0.168 | | | | | | | | | 0.004 | | | |
| Discrepancy → Neg | | | | | | | | 0.323 | | | | | | | | | < 0.001 | | | |
| Low → Anx | | | | | | | | 0.121 | | | | | | | | | < 0.001 | | | |
| Neg → Anx | | | | | | | | 0.655 | | | | | | | | | < 0.001 | | | |
| High Standards → Anx | | | | | | | | −0.053 | | | | | | | | | 0.271 | | | |
| Order → Anx | | | | | | | | −0.003 | | | | | | | | | 0.949 | | | |
| Discrepancy → Anx | | | | | | | | 0.122 | | | | | | | | | < 0.001 | | | |
| Note. Hybrid model fit: χ²(246) = 842.546; CFI = 0.941; TLI = 0.933; RMSEA = 0.059; SRMR = 0.044. The two perceived-stress components and anxiety symptoms were modeled as latent variables; APS-R subscale scores remained observed predictors. The residual covariance between the two perceived-stress factors was freely estimated. | | | | | | | | | | | | | | | | | | | | |
| **Panel D. Measurement invariance** | | | | | | | | | | | | | | | | | | | | |
| **Measure/grouping** | **Model** | **χ² (df)** | **CFI** | | | | **TLI** | | **RMSEA**  **(90% CI)** | | | | **SRMR** | **ΔCFI** | | | | **ΔRMSEA** | | **Decision** |
| PSS-14; Gender | Configural | 537.523 (152) | 0.925 | | | | 0.911 | | 0.060  [0.055, 0.066] | | | | 0.054 | — | | | | — | | Baseline |
|  | Metric | 550.685 (164) | 0.925 | | | | 0.917 | | 0.058  [0.053, 0.063] | | | | 0.059 | −0.000 | | | | −0.002 | | Supported |
|  | Scalar | 578.537 (176) | 0.922 | | | | 0.920 | | 0.057  [0.052, 0.062] | | | | 0.059 | −0.003 | | | | −0.001 | | Supported |
| PSS-14; Institution type | Configural | 585.791 (152) | 0.917 | | | | 0.900 | | 0.064  [0.058, 0.069] | | | | 0.056 | — | | | | — | | Baseline |
|  | Metric | 595.586 (164) | 0.917 | | | | 0.908 | | 0.061  [0.056, 0.066] | | | | 0.059 | +0.000 | | | | −0.003 | | Supported |
|  | Scalar | 636.083 (176) | 0.912 | | | | 0.909 | | 0.061  [0.056, 0.066] | | | | 0.060 | −0.005 | | | | −0.000 | | Supported |
| GAD-7; Gender | Configural | 93.446 (28) | 0.981 | | | | 0.972 | | 0.058  [0.045, 0.071] | | | | 0.024 | — | | | | — | | Baseline |
|  | Metric | 100.066 (34) | 0.981 | | | | 0.977 | | 0.053  [0.041, 0.065] | | | | 0.036 | −0.000 | | | | −0.005 | | Supported |
|  | Scalar | 110.559 (40) | 0.980 | | | | 0.979 | | 0.050  [0.039, 0.061] | | | | 0.036 | −0.001 | | | | −0.002 | | Supported |
| GAD-7; Institution type | Configural | 102.602 (28) | 0.978 | | | | 0.968 | | 0.062  [0.049, 0.075] | | | | 0.026 | — | | | | — | | Baseline |
|  | Metric | 107.177 (34) | 0.979 | | | | 0.974 | | 0.055  [0.044, 0.067] | | | | 0.034 | +0.000 | | | | −0.006 | | Supported |
|  | Scalar | 115.599 (40) | 0.978 | | | | 0.977 | | 0.052  [0.041, 0.063] | | | | 0.034 | −0.001 | | | | −0.003 | | Supported |
| **Note.** The adjusted model controlled for gender, year of study, institution type, and high-level university team membership and used HC3 heteroskedasticity-robust standard errors. Scalar invariance was supported when ΔCFI was no lower than −.010 and ΔRMSEA did not increase by more than .015. The analytic file did not retain case-level university identifiers; institution-clustered estimates were therefore not calculated. | | | | | | | | | | | | | | | | | | | | |
| **Panel E. Additional diagnostics** | | | | | | | | | | | | | | | | | | | | |
| **Analysis** | | | | **Result** | | | | | | | | | | | | **Interpretation** | | | | |
| Single-factor CFA across all psychological items | | | | χ²(902) = 12790.109; CFI = 0.324; TLI = 0.291; RMSEA = 0.137 [0.135, 0.139]; SRMR = 0.188 | | | | | | | | | | | | One common factor was insufficient; this does not rule out all shared-method influence | | | | |
| Age outlier sensitivity | | | | Excluding the participant aged 39 years left the focal directions and significance pattern unchanged | | | | | | | | | | | | No material influence on the conclusions | | | | |
| University clustering | | | | The analytic dataset contained institution type but no verified case-level university identifier | | | | | | | | | | | | ICCs and university-clustered standard errors could not be calculated without a case-to-university mapping | | | | |
| Note. Scalar invariance was considered supported when ΔCFI was no lower than −0.010 and ΔRMSEA did not increase by more than 0.015. The institution-level count sheet did not match the institution-type totals in the analytic dataset and could not be used to reconstruct individual university membership. | | | | | | | | | | | | | | | | | | | | |
